# Supplementary material for: Ab initio machine-learning unveils strong anharmonicity in non-Arrhenius self-diffusion of tungsten
Source: Nat Commun. 2025 Jan 4;16:394. doi: 10.1038/s41467-024-55759-w (PMC11700192; doi:10.1038/s41467-024-55759-w)
Supplement: Supplementary file 1 — Supplementary Information [file 41467_2024_55759_MOESM1_ESM.pdf]

Supplementary Information for "*Ab initio* machine-learning unveils strong anharmonicity in non-Arrhenius self-diffusion of tungsten"

Xi Zhang,[1] Sergiy V. Divinski,[2] and Blazej Grabowski[1]

[1] *Institute for Materials Science, University of Stuttgart, D-70569 Stuttgart, Germany*
[2] *Institute of Materials Physics, University of Münster, 48149 Münster, Germany*

Suppl. Note 1. Lattice expansion

Explicit calculations for $G_{\mathrm{form}}(T)$ and $G_{\mathrm{mig}}(T)$ were performed at eight temperatures starting at zero K and going to above the PBE melting temperature of 3349 K [1] (0 K, 600 K 1200 K, 1700 K, 2100 K, 2500 K, 3000 K, and 3400 K), followed by a fourth-order polynomial fit. For all calculations, the thermal lattice expansion effect was fully taken into account. The corresponding utilized equilibrium lattice constants are marked in Supplementary Figure 1(a) on the thermal expansion curves as red (DFT full) and gray (DFT qh) dots. The equilibrium 0 K lattice constant obtained in this work is 3.1723 Å, slightly larger than the experimental value of 3.16 Å. The relative lattice expansion is displayed in Supplementary Figure 1(b) in comparison to experimental data.

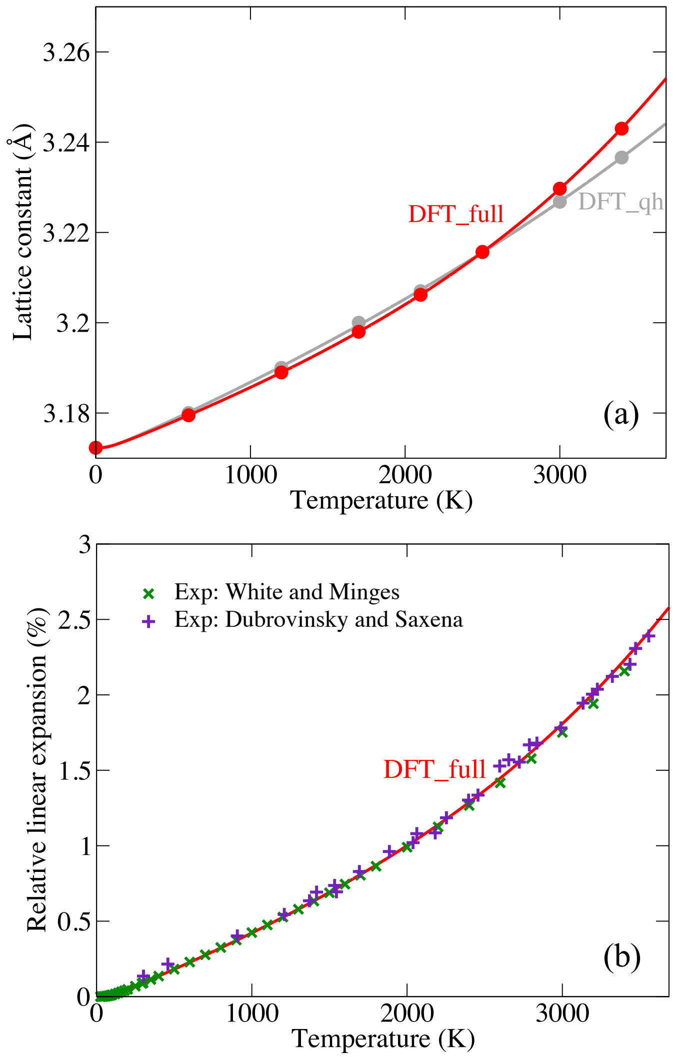

Supplementary Figure 1. (a) Temperature-dependent lattice constants used in this work. (b) Relative linear lattice expansion compared with experiments: White and Minges [2]; Dubrovinsky and Saxena [3].

Suppl. Note 2. Local environment in the MTP model

Supplementary Figure 2 illustrates the local atomic environment and the cutoff radius defined in the MTP model.

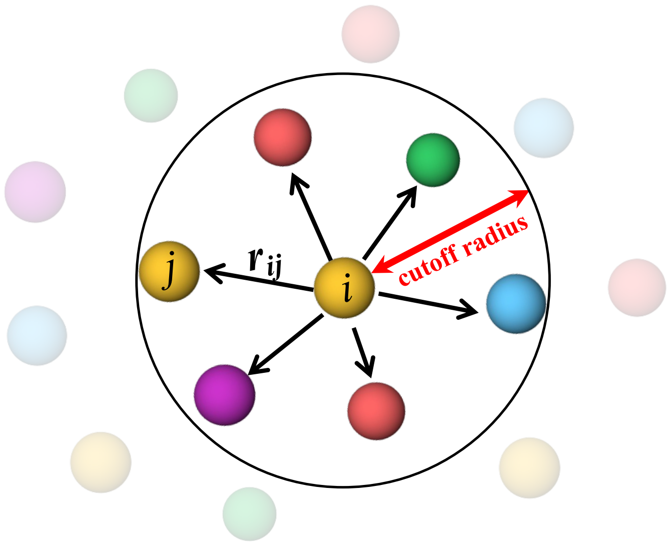

Supplementary Figure 2. Schematic illustration of the local environment in the MTP model.

Suppl. Note 3. Stabilization parameter in TSTI

To investigate the impact of the stabilization parameter α on the anharmonic free energy, we have tested a large range of α values from 0.5 to 100. Supplementary Figure 3 shows the change in the anharmonic free energy of the transition state (red circles) as a function of α (top x-axis), expressed on the bottom x-axis in terms of the resulting stabilized frequency. The representation in frequency has the benefit of relating the α values to the frequency spectrum of the transition state. The latter is represented by the phonon density of states (shown by the gray curve and shading). For α values up to a frequency of about 30 meV, the anharmonic free energy increases and then saturates, i.e., the α dependence reaches a plateau above the highest frequency in the system. It is important to note that the observed changes in F^{ah} are comparatively small ($\approx 1\%$) and close to the statistical resolution limit of the calculations, even when using a machine-learning potential. We conclude that the impact of the α parameter on the anharmonic free energy is rather small.

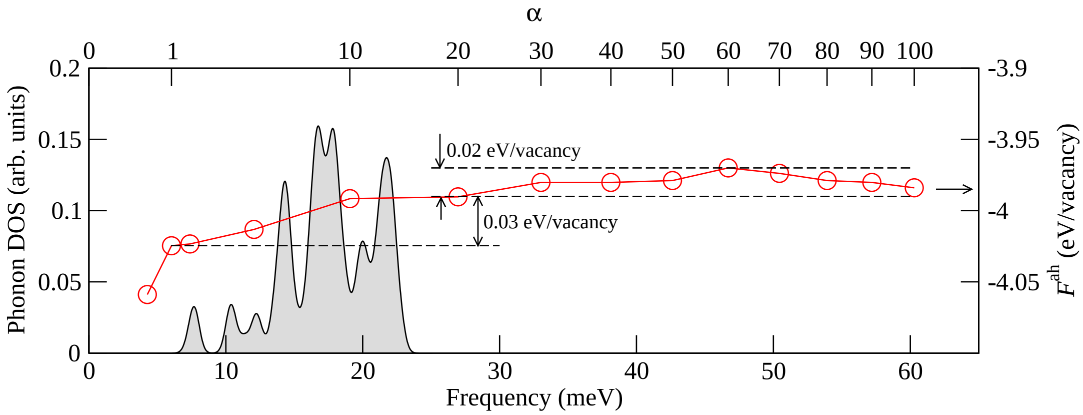

Supplementary Figure 3. Variation of the anharmonic free energy (red open circles) with different stabilized harmonic vibrational frequencies for the single unstable mode. The corresponding α values are marked on the top x-axis. The characteristic phonon density of states (DOS) for the $3N-1$ stable modes at the transition state is shown with gray shading.

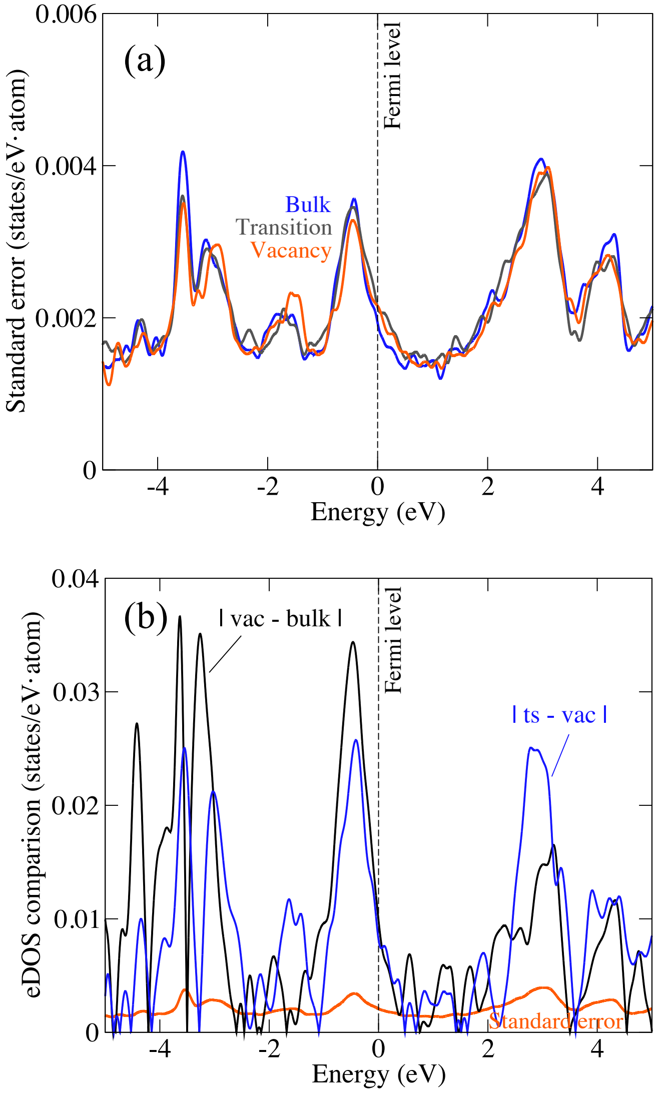

Supplementary Figure 4. Statistical analysis of the eDOS's: (a) standard errors and (b) comparison between the eDOS differences and the mean standard error.

We have also carefully tested the influence of α on the distributions shown in Figure 5 in the main text. The distributions involving the first nearest neighbor of the migrating atom are unaffected. The distributions involving the second nearest neighbor are unaffected along the $\mathbf{v_3}$ direction. In particular, the asymmetry introduced by anharmonic vibrations remains the same in absolute terms along $\mathbf{v_3}$. Along the $\mathbf{v_2}$ direction, both the anharmonic and harmonic distributions become narrower with increasing α. They converge to a small but finite width, and the asymmetry introduced by the anharmonic vibrations remains the same in relation to the harmonic distribution.

Suppl. Note 4. Electronic DOS convergence

To quantify the uncertainty of the mean eDOS's shown in Supplementary Figure 4 of the main text, we plot the standard errors in Figure 4(a) and compare them with the differences between different types of structures, i.e., $|\mathrm{eDOS_{bulk}}-\mathrm{eDOS_{vac}}|$ and $|\mathrm{eDOS_{vac}}-\mathrm{eDOS_{ts}}|$, in Supplementary Figure 4(b). While the general features in terms of peaks and valleys between the standard error and the differences are similar, the values are about one order of magnitude smaller in particular at the valleys and peaks of the eDOS's where the differences shows a peak. This means the mean eDOS's averaged over 120 MD snapshots shown in Supplementary Figure 4 of the main text are well converged.

Suppl. Note 5. Diffusivity on the absolute temperature scale

On the absolute temperature scale, while the predicted diffusivity is about two orders of magnitute higher than the experimental data, the general temperature dependence shows a good agreement. We attribute the discrepancy in magnitude to the employed PBE exchange-correlation functional which underestimates the melting point of W by more than 300 K [1].

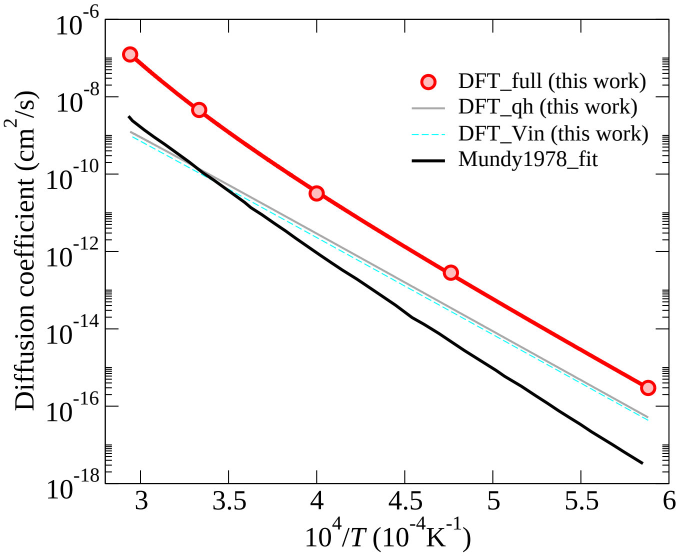

Supplementary Figure 5. Arrhenius plot of self-diffusivity in bcc tungsten calculated with the proposed *ab initio* machine-learning TSTI approach with all finite-temperature excitations taken into account (red circles and lines) on the absolute temperature scale in comparison to the fitted experimental data from Mundy et al. [4].

Suppl. Note 6. Uncertainty analysis

Results of the uncertainty estimation of the target quatities are summarized in Supplementary Table 1. We focus on the propagation of the statistical uncertainty in each free energy contribution to estimate the total uncertainty of the calculated formation and migration Gibbs energies as well as diffusivities. For each free energy value at the given temperature, following the concept of the Student's t-distribution, the uncertainty δ is calculated as

$$\delta = t_{95\%,n-1}\frac{\sigma}{\sqrt{n}}, \tag{S1}$$

where σ and n refer to the standard deviation and the sample size and $t_{95\%,n-1}$ is the two-sided t value of the 95% confidence with $n-1$ degrees of freedom. According to the propagation of the statistical uncertainty, the total uncertainty of a function of $A \pm B \pm ...$ is calculated as $\sqrt{\delta_A^2 + \delta_B^2 + ...}$, so the uncertainty of the diffusivity can be estimated as

$$\delta_{\text{diff}} = D(T)\sqrt{\delta_{\text{form}}^2 + \delta_{\text{mig}}^2}/(k_B T), \tag{S2}$$

where $D(T)$ is the calculated diffusivity and δ_{form} and δ_{form} are the uncertainty of the formation and migration Gibbs energy, respectively.

Suppl. Note 7. On the di-vacancy hypothesis

In a previous high-accuracy DFT study [5], the di-vacancy mechanism has been convincingly ruled out in FCC elements (i.e., Al and Cu) by explicit finite-temperature DFT calculations of di-vacancy formation Gibbs energies. Therein, clear contradictions with experimental observations in terms of the vacancy concentration and the formation

Supplementary Table 1. Estimation of the uncertainties. All energy uncertainties are in units of eV/cell.

T	Bulk				Vacancy				Transition state				$G_{\rm form}$	$G_{\rm mig}$	D
(K)	$\Delta F^{\rm qh\rightarrow full}$	$\Delta F^{\rm up}$	$F^{\rm el}$	Total	$\Delta F^{\rm qh\rightarrow full}$	$\Delta F^{\rm up}$	$F^{\rm el}$	Total	$\Delta F^{\rm qh\rightarrow full}$	$\Delta F^{\rm up}$	$F^{\rm el}$	Total			(cm^2/s)
600	0	0.004	0.001	0.004	0	0.005	0.001	0.005	0	0.01	0.001	0.01	0.007	0.012	-
1200	0.002	0.008	0.006	0.01	0	0.015	0.005	0.016	0.001	0.01	0.005	0.012	0.02	0.02	-
1700	0.005	0.014	0.009	0.017	0.006	0.01	0.01	0.017	0.004	0.01	0.014	0.02	0.024	0.026	7.2×10^{-17}
2100	0.01	0.01	0.017	0.023	0.02	0.012	0.016	0.028	0.008	0.01	0.017	0.022	0.036	0.035	7.9×10^{-14}
2500	0.02	0.014	0.022	0.033	0.013	0.018	0.022	0.031	0.03	0.016	0.026	0.04	0.045	0.052	1.0×10^{-11}
3000	0.024	0.015	0.026	0.038	0.013	0.016	0.029	0.036	0.02	0.016	0.03	0.04	0.052	0.053	1.3×10^{-9}
3400	0.013	0.019	0.04	0.046	0.004	0.02	0.034	0.04	0.03	0.024	0.038	0.055	0.06	0.068	3.8×10^{-8}

entropy were obtained for the di-vacancy hypothesis. For BCC refractory elements like tungsten, due to the significant challenges in experimental measurements of mono- and di-vacancy concentrations at very high temperatures, the same set of arguments as given in Ref. [5] for FCC elements cannot be directly utilized. We thus provide here an alternative analysis based on experimental diffusion data and the available monovacancy and di-vacancy energetics to substantiate that di-vacancies can hardly explain the non-Arrhenius diffusion of BCC W.

By assuming a collective mechanism of diffusion (i.e., mono- and di-vacancies) in BCC W, the diffusivity can be expressed as:

$$D = \frac{c_{1v}}{c_{1v}+2c_{2v}}D_{1v} + \frac{2c_{2v}}{c_{1v}+2c_{2v}}D_{2v} \tag{S3}$$

$$= f_1 D_{1v,0}\exp\left(-\frac{Q_{1v}}{k_{\rm B}T}\right) + f_2 D_{2v,0}\exp\left(-\frac{Q_{2v}}{k_{\rm B}T}\right), \tag{S4}$$

where c_i and D_i ($i = 1v$ or $2v$) refer to the concentration of monovacancies ($1v$) or di-vacancies ($2v$) and their diffusivity contributions, respectively. Equation (S3) can be rewritten as shown in Eq. (S4), i.e., in terms of the fraction f_i of each type i of vacancy, the pre-factor $D_{i,0}$, and the activation energy Q_i. Fitting the measured diffusivities to Eq. (S4), as shown in Ref [4], results in (in cm^2/s):

$$D = 0.04\ \exp\left(-\frac{5.45\ \text{eV}}{k_{\rm B}T}\right) + 46\ \exp\left(-\frac{6.9\ \text{eV}}{k_{\rm B}T}\right), \tag{S5}$$

where the first term would correspond to the mono-vacancy diffusion since the activation energy (5.45 eV) agrees well with the sum of the mean values of the available experimental data for the mono-vacancy formation energy (3.8 eV) and migration energy (1.7 eV). It must be then that the second term would correspond to di-vacancy diffusion; however, in such a case a clear contradiction can be derived as follows.

The activation energy of the di-vacancy diffusion Q_{2v} can be written in terms of the mono-vacancy formation energy $H^{1v}_{\rm form}$, the binding energy of a vacancy pair $H^{2v}_{\rm bind}$, and the di-vacancy migration energy $H^{2v}_{\rm mig}$ as

$$Q_{2v} = 2H^{1v}_{\rm form} - H^{2v}_{\rm bind} + H^{2v}_{\rm mig}. \tag{S6}$$

The di-vacancy binding energy then reads

$$H^{2v}_{\rm bind} = 2H^{1v}_{\rm form} + H^{2v}_{\rm mig} - Q_{2v}. \tag{S7}$$

The experimental di-vacancy migration energy was reported by Park et al. [6] and by Rasch et al. [7] to be very similar to the mono-vacancy migration energy, i.e., about 1.8 eV. This was also confirmed by a recent DFT-based kinetic Monte Carlo study [8] where an effective di-vacancy migration energy of 1.65 eV was obtained. Based on these experimental mono-/di-vacancy energies together with the di-vacancy diffusion activation energy reported by Mundy et al. [4], the di-vacancy binding energy can be calculated as

$$\begin{aligned} H^{2v}_{\rm bind} &= 2H^{1v}_{\rm form} + H^{2v}_{\rm mig} - Q_{2v} \\ &= 2\times 3.8\ \text{eV} + 1.8\ \text{eV} - 6.9\ \text{eV} = 2.5\ \text{eV}. \end{aligned} \tag{S8}$$

Now, the value of $H^{2v}_{\rm bind} = 2.5$ eV is, however, dramatically larger than directly obtained theoretical and experimental values: DFT studies have shown very small di-vacancy binding energies ranging from -0.1 eV to $+0.05$ eV (see Ref. [8]). With our own DFT calculations, we also obtained a small 0 K binding energy of -0.01 eV. The only available experimental value is 0.7 eV, reported by Park et al. [6], which is still well below 1 eV. Such a large

discrepancy with respect to the value in Eq. (S8) cannot be explained by uncertainty. Therefore, the di-vacancy diffusion mechanism cannot be used to explain the non-Arrhenius diffusion of BCC W, or at least it must be that it only plays a minor role.

In fact, the consistent curvature of the full DFT results with experiment obtained in the present study based on a single mono-vacancy mechanism is clear and convincing evidence that the di-vacancy mechanism contribution is not appropriate in explaining the non-Arrhenius dependence.

Suppl. Note 8. Migration pathway at high temperatures

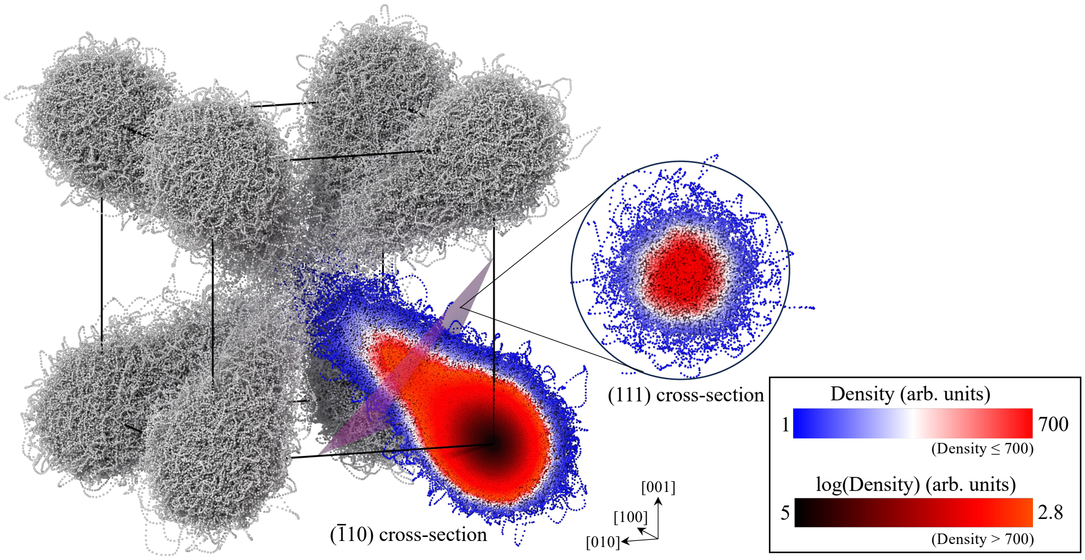

Supplementary Figure 6. Statistical plot of 754 migration pathways for BCC W collected from MTP-MD at 3400 K. The color code shows the density of the distribution. The densities (> 700) at the core of the distribution are plotted using their logarithmic values.

Supplementary Figure 6 depicts 754 migration pathways collected from MTP-MD runs at 3400 K (the highest temperature considered in this work) where the gray dots indicate the trajectories of atoms at the BCC corners vibrating and migrating toward the vacancy in the middle of the BCC unit cell (cube with the black edges). It is apparent that, on average, the migration pathways of the eight nearest neighbors of the vacancy preserve the full BCC crystalline symmetry, migrating along the $\langle 111 \rangle$ direction which is the same as for the NEB path at 0 K.

Moreover, we show the distribution density at the right bottom corner by cross-sections cut by the (110) plane and the (111) plane. The resulting statistical Gaussian distributions are centered along the [111] direction, thus further supporting that at high temperatures the migration pathways follow the 0 K NEB pathway within an ensemble average. This is due to the fact that for a symmetrical crystalline structure like BCC, the full symmetry needs to be preserved even at high temperatures. The distribution density shown in Supplementary Figure 6 agrees well with the description of the finite temperature string method that the finite-temperature trajectories can be described as a tube in configuration space. The tube center gives the reaction coordinate and results from the average over all trajectories.

Suppl. Note 9. Direct MTP MD simulations

In order to further validate the TSTI approach, for three selected high temperatures, i.e., 3000 K, 3200 K, and 3400 K, we performed explicitly direct MD simulations using the optimized MTP, from which the self-diffusivity of W can be extracted via

$$D = \frac{\langle R^2 \rangle}{6t} \tag{S9}$$

where $\langle R^2 \rangle$ is the mean squared displacement of the diffusing atom and t is the time.

MTP MD simulations were performed with LAMMPS [9] utilizing a simulation box comprised of 1024 atoms ($8 \times 8 \times 8$ expansion of the BCC unit cell) where a single vacancy was introduced. The DFT-predicted expanded lattice constants were used. For each selected temperature, 50 independent NVT MD runs were carried out for 20 ns using the Nose-Hoover thermostat with a damping parameter of 100 fs. The time step was set to be 1 fs.

The mean squared displacement as a function of time obtained at 3400 K is shown in Supplementary Figure 7 as an example. The resulting diffusivities from direct MTP MD simulations are shown in Supplementary Figure 8 and compared with the MTP TSTI results. Both are rescaled by the DFT-predicted vacancy concentrations at the selected temperatures by assuming that diffusivities are in proportion to the vacancy concentration. It is clear that diffusivities computed from both methods agree very well not only in the absolute values but also in the temperature dependence. This highlights the validity of the present TSTI computational framework and its high predictive power and accuracy.

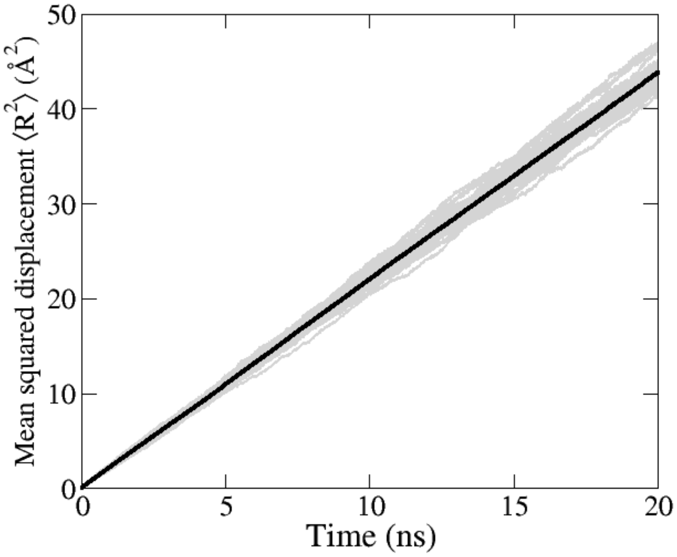

Supplementary Figure 7. Mean squared displacement as a function of time at 3400 K. Gray lines are the results of each independent MD run (50 in total) and the black line shows the mean values.

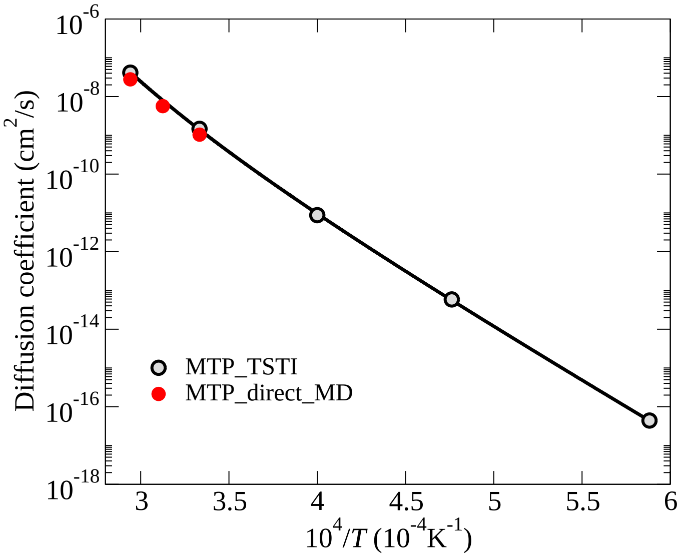

Supplementary Figure 8. Arrhenius plot of self-diffusivity computed from the present TSTI approach and direct MD simulations using MTP.

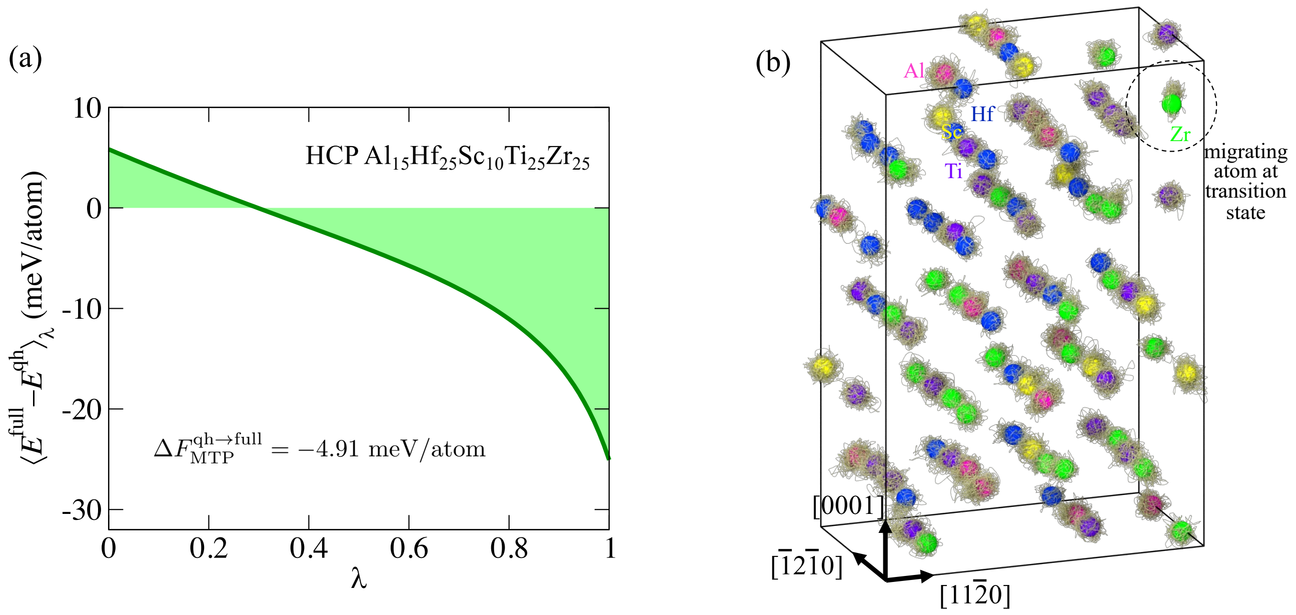

Supplementary Figure 9. (a) $\langle E^{\rm full} - E^{\rm qh} \rangle_\lambda$ as a function of λ for Zr diffusion at the transition state in an HCP $Al_{15}Hf_{25}Sc_{10}Ti_{25}Zr_{25}$ HEA calculated at 1000 K. Integration of the green line (green shaded area) gives the anharmonic free energy (with MTP accuracy) shown in the figure. (b) Illustration of the stable MD trajectory (silver lines; 10000 steps).

Suppl. Note 10. Application of TSTI in high-entropy alloys

First TSTI results for a five-component HCP high entropy alloy (HEA), $Al_{15}Hf_{25}Sc_{10}Ti_{25}Zr_{25}$, are shown in Supplementary Figure 9. The HEA was modeled with a special quasirandom structure with 95 atoms (4×4×3 expansion of the conventional HCP unit cell; cf. Supplementary Figure 9(b)). The anharmonic free energy of a Zr atom at the transition state (encircled in the figure) was investigated with TSTI at 1000 K. Specifically, the full λ dependence of $\langle E^{\rm full} - E^{\rm qh} \rangle_\lambda$ from a quasiharmonic reference to an MTP (RMSE of 2.5 meV/atom) was calculated and is plotted in Supplementary Figure 9(a). All transition-state trajectories entering the calculation are stable and the resulting curve is well-behaved. These results substantiate the robust performance of the TSTI approach also for chemically complex materials.

Supplementary References

[1] L.-F. Zhu, P. Srinivasan, Y. Gong, T. Hickel, B. Grabowski, F. Körmann, and J. Neugebauer, Melting properties of the refractory metals V and W and the binary VW alloy fully from first principles, Phys. Rev. B **109**, 094110 (2024).

[2] G. K. White and M. L. Minges, Thermophysical properties of some key solids: An update, International Journal of Thermophysics **18**, 1269 (1997).

[3] L. S. Dubrovinsky and S. K. Saxena, Thermal Expansion of Periclase (MgO) and Tungsten (W) to Melting Temperatures, Physics and Chemistry of Minerals **24**, 547 (1997).

[4] J. N. Mundy, S. J. Rothman, N. Q. Lam, H. A. Hoff, and L. J. Nowicki, Self-diffusion in tungsten, Physical Review B **18**, 6566 (1978).

[5] A. Glensk, B. Grabowski, T. Hickel, and J. Neugebauer, Breakdown of the Arrhenius Law in Describing Vacancy Formation Energies: The Importance of Local Anharmonicity Revealed by Ab initio Thermodynamics, Physical Review X **4**, 011018 (2014).

[6] J. Y. Park, H. C. W. Huang, R. W. Siegel, and R. W. Balluffi, A quantitative study of vacancy defects in quenched tungsten by combined field-ion microscopy and electrical resistometry, Philosophical Magazine A **48**, 397 (1983).

[7] K.-D. Rasch, R. W. Siegel, and H. Schultz, Quenching and recovery investigations of vacancies in tungsten, Philosophical Magazine A **41**, 91 (1980).

[8] K. Heinola, F. Djurabekova, and T. Ahlgren, On the stability and mobility of di-vacancies in tungsten, Nuclear Fusion **58**, 026004 (2017).

[9] A. P. Thompson, H. M. Aktulga, R. Berger, D. S. Bolintineanu, W. M. Brown, P. S. Crozier, P. J. in 't Veld, A. Kohlmeyer, S. G. Moore, T. D. Nguyen, R. Shan, M. J. Stevens, J. Tranchida, C. Trott, and S. J. Plimpton, LAMMPS - a flexible simulation tool for particle-based materials modeling at the atomic, meso, and continuum scales, Comp. Phys. Comm. **271**, 108171 (2022).
